# Some of the Papers

**Published:** 1907-08-10

**Authors:** 


### THE BRITISH MEDICAL ASSOCIATION AT EXETER.

### SOME OF THE PAPERS.

On Wednesday, July 31, the active work of the sectional meetings commenced. The first demonstration was held in the lecture room at the Barnfield Hall, where a large number of members attended to inspect the excellent collection of pathological specimens, and in various parts of the town the sectional meetings were held.

### Archæological Expeditions.

Though the sectional meetings were, on the whole, well attended, a large number of members preferred the excursions and rambling parties to the indoor demonstrations that invited their attention. So the excursions organised by some enthusiastic members to inspect the town and its archæological curiosities attracted quite a large number of members, and those who took part in these enjoyable rambles will look back with a great deal of pleasure to the few hours they spent under the guidance of Mr. Reed and Chancellor Edwards.

### The Pathology of Pernicious Anæmia.

In the pathological section Dr. William Hunter, whose name is already well known in connection with his work on the etiology of ganglionic anæmia, opened the discussion on this interesting disease. He started by briefly sketching the history of the disease, and went on to discuss our conceptions of the pathology of the condition, venturing to express the opinion that the causative factor was a deficiency in blood-forming power. He gave particulars of some cases which had improved under vaccine treatment, and drew attention to the remarkable series of hæmolytic changes observable in the viscera. A fine collection of illustrative specimens was shown, enabling members to follow the discussion to much better advantage. In the discussion that followed some interesting references were made to treatment. Dr. Gullan detailed two cases in which there had been profound anæmia of a pernicious type, and in which rapid recovery had followed systematic feeding by bone-marrow preparations. Dr. Bushnell reported that he had so far experienced no striking improvement in cases of anæmia treated with streptococcal vaccine.

### Indications for Operation in Cases of Brain Tumour.

At the first meeting of the section of medicine Dr. Risien Russell initiated the discussion on the above subject. He emphasised the necessity for early diagnosis of intracranial tumour, and appealed for early surgical interference in cases where the diagnosis had been made, and where the case was deemed suitable for operation. He laid stress on the difficulties which sometimes attend the differential diagnosis of intracranial tumours, and on the uncertainty of diagnosing the nature of the tumour before operation. The position of the growth, he thought, was of the greatest importance in considering the question of active surgical interference. A tumour located deep in the hemispheres, in the mesencephalon itself, or in the middle lobe of the cerebellum, could scarcely be successfully dealt with by operation. He advocated operation in cases of syphilitic tumours where no marked improvement had followed medical treatment. With this opinion Sir William Macewen cordially agreed, pointing out that operation was sometimes advisable in cases where the diagnosis was obscure, and where the classical symptoms of brain tumour were not present. Gummatous tumours, if large and probably caseating, called for surgical interference; and drug treatment, if it had yielded no striking improvement after having been tried for a reasonable length of time, should not be persisted in. Dr. Pitt also thought that cerebral gummata could not be entirely removed by drug treatment. Professor Osler was of opinion that drug treatment held out a reasonable chance of cure in cases of cerebral gummata, and thought that there was ground for the prevailing scepticism of the profession with reference to the successful removal of intracranial tumours. Operation in such cases called for a special degree of skill on the part of the surgeon, and a very careful consideration of the features of each individual case.

### Alcohol and Insanity.

In the section devoted to the discussion of psychological medicine Dr. Mott read a paper on alcohol and insanity, in which he communicated the results of a series of observations on hospital and asylum patients. Cirrhosis of the liver was comparatively rare in asylum patients, though common enough among hospital patients. His main deduction from his study of a large number of cases was that insanity due to alcohol was much rarer than official tables would lead one to believe, and that where it did occur the patients were "already the subjects of a locus minoris resistentiæ," and other causative factors had already been at work. In the majority of cases alcohol did not cause a definite mental derangement to which the term insanity could be applied. An interesting discussion resulted, the majority of those taking part in it agreeing with the author of the paper that alcoholic insanity was comparatively rare. Dr. Macdonald, for instance, pointed out that in districts where the ratio of insanity to population was relatively high, insanity due to drink was comparatively low, as in Lancashire and Durham. Professor Anderson pointed out how limited in reality was our knowledge of alcohol, and how probable it was that the by-products and essential oils might exercise great influence upon particular cases. Among those who differed from Dr. Mott were Drs. Robertson and Oswald, who contended that alcohol was one of the greatest factors in the production of insanity, and that delirium tremens was one of the most certain and typical of all the insanities.

### The Temperance Breakfast.

On Thursday morning the Science and Education Committee of the National Temperance League gave a breakfast in the Royal Public Rooms, the Mayor of Exeter occupying the chair. Dr. Davy, the President of the Association, in the course of a short speech, said that the temperance question was one which had a great deal of interest for medical men. They could not but consider that the enormous annual expenditure on alcohol was a serious matter, especially when it was realised that alcohol was responsible for much of the crime and sickness to be observed. At the same time most total abstainers and temperance advocates talked the most unscientific twaddle. It was nonsense to tell children that if they drank a glass of wine they were doing a moral wrong, or saturating themselves with poison. Personally he thought that a meal of bread, cheese, and light beer was infinitely more scientific, and therefore better, than one of bread, tea, and jam.

### The Annual Luncheons.

Thursday was the day of "reunions." At the Rougemont Hotel the Irish Medical Schools and Graduates' Association foregathered, under the genial chairmanship of Sir John Moore. At the New London Hotel Professor Osler presided over the luncheon of the Continental Anglo-American Medical Society, a function which was attended by many guests and visitors.

### General Anæsthesia.

In the section of surgery the secretary, in the absence of the author, communicated Mr. Dean's paper on the relative value of injection and inhalation methods of inducing general anæsthesia. The author strongly advocated the claims of lumbar injection for producing anæsthesia, pointing out that shock was still a considerable factor to be reckoned with in ordinary inhalation anæsthesia, induced by chloroform or ether vapour. Both these drugs acted on the central nervous system, while in the so-called "lumbar method" the anæsthetist possessed a means of safeguarding the higher centres from pernicious peripheral stimuli. The author recommended stovaine, as possessing distinct advantages over cocaine for lumbar injections to produce anæsthesia, and discussed the relative merits of other preparations, including novocaine and alypin. He particularly favoured lumbar anæsthesia in cases where surgical shock was anticipated—as, for instance, in acute general peritonitis. In the discussion that ensued members were of opinion that sufficient statistical data not being available it was impossible to estimate accurately the relative values of the various methods. Dr. Wilson, speaking as an anæsthetist, thought that chloroform and ether narcosis, in proper hands, were really quite safe, and that in the majority of abdominal operations surgical shock could be efficiently guarded against. Mr. Leedham-Green said he used injection anæsthesia as a routine method, and warmly advocated the employment of tropococaine, which was safe, reliable, and produced no undesirable after-effects. Dr. Levy, who supported the open method of ether administration, thought that the disadvantages of inhalation methods had been exaggerated. Professor Rodman, of Philadelphia, detailed his experience of injection methods in cases of bronchitis, nephritis, cardiac disease, and alcoholism, and stated that he thought it was unsuitable for operations on the upper part of the abdomen, and that it should not be used in operations on children.

### The Differential Diagnosis of Some Laryngeal Lesions.

Sir Felix Semon read an interesting paper on the differential diagnosis between tuberculous, syphilitic, and malignant disease of the larynx, at the first meeting of the section devoted to laryngology and rhinology. Usually the diagnosis was comparatively easy, but under some conditions a definite diagnosis was almost an impossibility. He laid great stress on a unilateral congestion of the vocal cords, which he thought was a diagnostic point of great value. Tuberculous growths sometimes occurred when there was no demonstrable pulmonary lesion, and sometimes in old patients. Examination of the sputum and microscopical examination of a portion of the growth excised for that purpose, were important aids towards establishing a diagnosis. Formerly it was held that tubercle and malignant disease of the larynx were mutually exclusive, but that view was no longer tenable, and the two conditions might co-exist. In every case of doubt he advocated a trial of antisyphilitic treatment. In the discussion that followed several members took part. Dr. Horne, in a paper dealing with ambiguous cases, recognised four stages: congestion and impairment of vocal function, swelling, ulceration, and involvement of glands. He thought a diagnosis might be arrived at by a process of careful elimination, in which the data furnished by microscopical examination of the excised portion, by an estimation of the patient's opsonic index, and by a very careful laryngological inspection, would be helpful.

### Ophthalmia Neonatorum.

In the ophthalmological section several interesting papers were read. Professor Landolt, of Paris, gave a demonstration of instruments, exhibiting several new types, for which he himself was responsible. Mr. Snell gave a summary of his study of 300 cases of blindness. All these were in inmates of a blind school, and in more than a third the direct cause of the blindness was ophthalmia neonatorum, the other causative factors being optic atrophy, sympathetic ophthalmitis, and congenital cataract. A resolution was tabled "That, in the opinion of this section, the time has come for the British Medical Association to take action for the prevention of ophthalmia neonatorum." Members, in discussing the resolution, referred to the increasing prevalence of the disease. In Paris, Professor Landolt remarked, ophthalmia neonatorum was not so common nowadays as it had been. Dr. Hern, of Darlington, on the other hand, stated that in his district it was increasing, and most of the cases he saw were in patients who had been treated by midwives. Mr. Snell stated that the disease was not decreasing in Sheffield. The resolution was passed unanimously.

